# Correlation of revision rate of unicompartmental knee arthroplasty with total knee arthroplasty: a meta-analysis of clinical studies and worldwide arthroplasty registers

**DOI:** 10.1007/s00402-024-05574-1

**Published:** 2024-10-15

**Authors:** Stephan Obermayr, Antonio Klasan, Laura Rasic, Georg Hauer, Lukas Leitner, Andreas Leithner, Patrick Sadoghi

**Affiliations:** 1 Department of Orthopedics and Trauma, Medical University of Graz, Auenbruggerplatz 5, 8036 Graz, Austria; 2 AUVA UKH Steiermark, Graz, Austria; 3 Johannes Kepler University Linz, Linz, Austria; 4 Department of Orthopedics and Trauma Surgery, Musculoskeletal University Center Munich (MUM), LMU University Hospital, Munich, Germany

**Keywords:** Unicompartmental knee arthroplasty, Total knee arthroplasty, Arthroplasty register, Revision rate, Systematic review

## Abstract

**Introduction:**

The purpose of this study was to elucidate differences and similarities in revision rates amongst studies and national registers featuring total knee arthroplasty (TKA) and unicompartmental knee arthroplasty (UKA). Thereby comparability and reproducibility between study and register findings should be created.

**Materials and Methods:**

Clinical studies published between 2004 and September 2023 involving TKA or UKA were reviewed for total arthroplasty numbers, revision rates and demographic data. Findings were calculated as “revisions per 100 component years (CY)” and divided according to the nationality of the center. National arthroplasty registers were searched for numbers of arthroplasties and revisions alongside with demographic data. Revision rates in registers were compared to one another and comparison to revision rates from collected studies was drawn.

**Results:**

After evaluation, 98 studies and seven registers met our inclusion criteria and were included in this study. Cumulative percent revision rate in studies was 3.35% after a mean follow-up of 5.7 years, corresponding to 0.71 revisions per 100 CY for TKA and 7.67% after a mean follow-up of 4.9 years, corresponding to 1.3 revisions per 100 CY for UKA. Registers showed mean overall revision rates of 5.63% for TKA and 11.04% for UKA.

**Conclusions:**

A positive correlation of revision rates of TKA and UKA in studies and registers was found, with overall revision rates of UKA comparted to TKA being 2.29 times higher in clinical studies and 1.96 times higher in registers. Revision rates in registers were 1.56 times higher than presented in clinical studies.

## Introduction

Total knee arthroplasty (TKA) and unicompartmental knee arthroplasty (UKA) provide effective, rapid, and economical methods in restoring physiological articular function, reducing pain, and improving the quality of life in patients with severe osteoarthritis (OA) [1–3]. The prevalence of OA is steadily increasing as the obesity epidemic and aging of the population is gaining traction [2, 4]. If the OA is limited to one compartment, UKA enables the treatment of exclusively the medial or lateral compartment, while TKA is commonly used in knees, where the affected area is greater than one compartment [5, 6]. Unicompartmental knee arthroplasties show less mortality, morbidity and blood loss due to less resection of tissue, while restoring the normal kinematics of the joint by maintaining the function of the cruciate ligaments, resulting in superior functional outcome in comparison to TKA [5, 7]. Besides, patients receiving UKA being younger and more active, the recovery period and duration of hospitalization is shorter and major complications were registered less frequently, which results in lower cost in respect to TKA [5, 7].

Revision surgery of knee arthroplasty is scarse, but has severe consequences for the quality of life of the patient [5, 8, 9]. Most common reasons for revision surgery are infection, aseptic loosening, periprosthetic fracture, instability, pain, arthrofibrosis, polyethylene wear, patella failure or implant failure [8, 10, 11]. Higher revision rates in UKA compared to TKA is not linked to the younger age of patients receiving UKA or poorer outcome, but lower threshold considering revision surgery, which will typically result in a TKA [5, 12, 13]. Furthermore surgeons who perform less UKA tend to have higher revision rates [5, 7]. While clinical studies present a small part of the patient population, national registers include all surgeries performed in a country. To provide further assessment of revision rates of TKA and UKA and compare clinical results amongst countries, it is vital to draw comparisons between clinical studies and national registers and among one another [14, 15].

The aim of this meta-analysis was to evaluate, whether revision rates of TKA and UKA reported in clinical studies and national arthroplasty registers show potential correlation amongst different regions. Our hypothesis was, that regions presenting higher revision rates for TKA and UKA in registries, also show relatively high revision rates in studies.

## Methods

### Search strategy

A systematic research concerning revision rates of UKA and TKA was performed consulting Embase, PubMed and the Cochrane Controlled Trials Registry using the search terms: “(“unicompartmental knee arthroplasty” OR “total knee arthroplasty”) AND (“revision rates” OR “survival rates”)” [16, 17]. After titles and abstracts were reviewed by hand, we analyzed full text studies and noted relevant information in September 2023. References of included studies were screened for articles who could provide further case numbers and were excluded by our search term. Upon request, the complete results including our absolute research algorithm will be available. The PRISMA guideline (Preferred Reporting Items for Systematic Reviews and Meta-analyses) was used to ensure the highest quality of results [18].

### Inclusion/exclusion

Inclusion criteria were clinical studies covering TKA and UKA with publication dates between 01.01.2004 and 11.09.2023. The follow-up time had to exceed 12 months and revision rates and reasons for revision had to be mentioned or could be calculated from the provided data. Revision surgery was defined as the removal, addition, or replacement of at least one component of the implanted prothesis or reoperation due to linked complications. Furthermore, information regarding geographical location of the medical center, component design, fixation type, mean age and sex had to be stated. Only papers published in peer-reviewed journals published in English or German language were examined. Comparing multiple studies treating the same cohort, the trial with the longest follow-up-period was included. Cadaveric studies, reviews, meta-analyses, imaging studies and case reports, along with trails showing less than a 12-month follow-up period were excluded. Studies reporting on data from private registers were included.

### Quality assessment

All national arthroplasty registers and studies were searched by the author and co-author (S.O. and L.R.) independently to prevent bias. The Levels of Evidence according to the Oxford Centre for Evidence-Based Medicine were assessed when provided [19].

### Data extraction

Every study that fulfilled the inclusion criteria was independently reviewed in full-text by the authors (S.O. and L.R.). The following data was noted: name of the article, PMID, DOI, authors, year of publication, country of publication, total UKA and TKA, distribution of sex, mean age, mean follow-up, number of revisions UKA/TKA and reasons for revision. Further calculations were made using this information. The process was supervised by the senior author who resolved any disagreements on consensus discussions between authors. Missing absolute revision numbers were calculated when possible.

### Acquirement of clinical studies

**Fig. 1:**
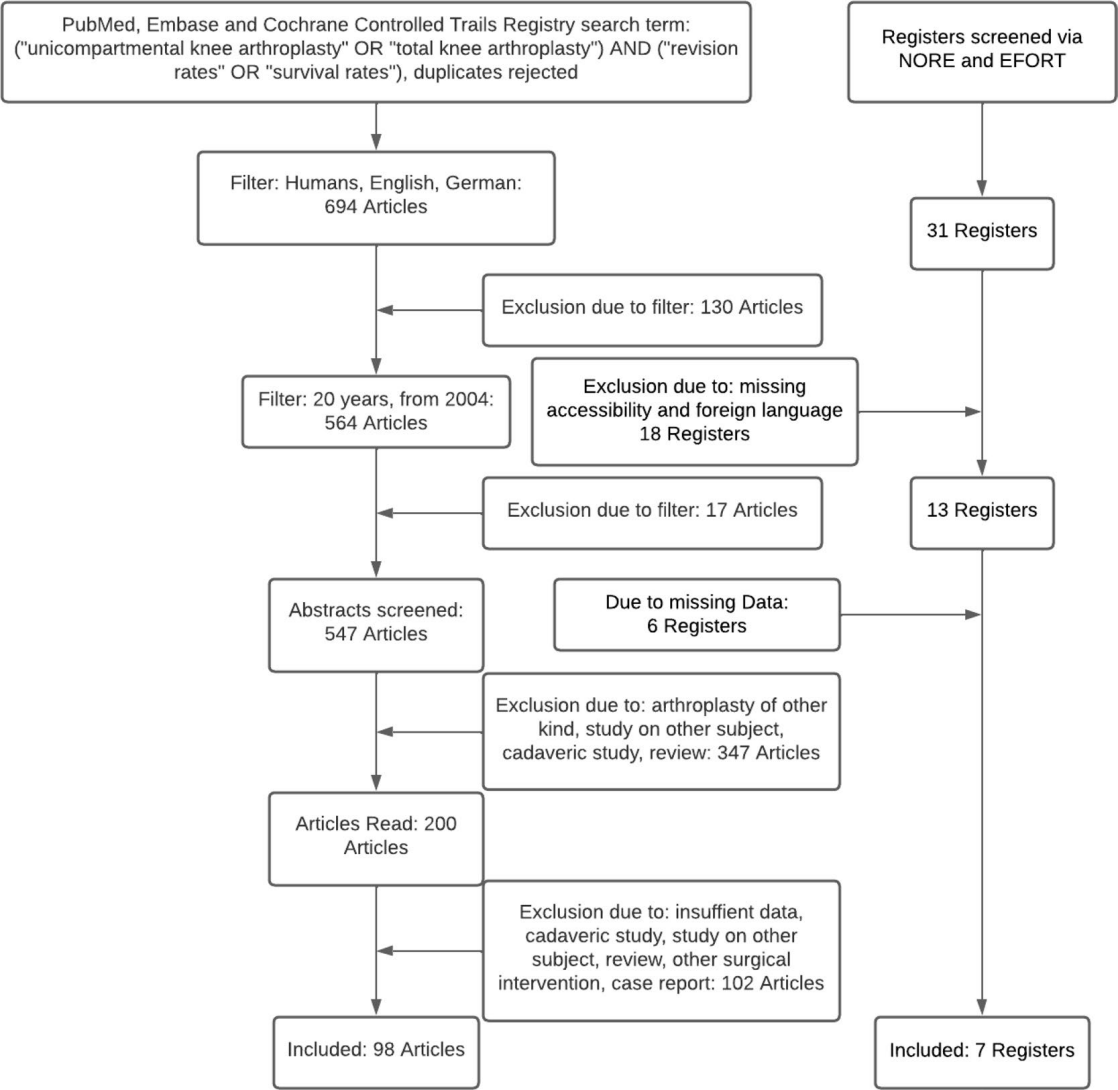
Search process and acquirement of clinical studies and national registers

Our search algorithm revealed 547 articles, of which 347 articles were excluded after a screening of title and abstract and application of inclusion and exclusion criteria. A total of 200 studies were evaluated as a whole. Finally, 98 articles met our inclusion and exclusion criteria and were used for assessment (Fig. 1).

### Clinical studies on revision rates of total knee arthroplasty

There were 461,387 total knee arthroplasties from 72 articles, covering a total of 2,695,243.4 observed CY. Studies included in our review were published from 2005 to 2023 (Table 1).

**Table 1:** Data of clinical studies portraying total knee arthroplasty

Authors	Year of publication	Country	Total TKA (n)	Mean Age (y)	Mean follow-up (y)	Revisions TKA (n)
Katchky et al.[20]	2019	Australia	100	68	5.3	2
Klasan et al.[21]	2019	Australia	1288	76.6	7.8	13
Hazratwala et al.[22]	2023	Australia	165	65.1	2	3
Victor et al.[23]	2014	Belgium	245	68.1	11	11
Beaupré et al.[24]	2007	Canada	81	63.4	5	2
MacDonald et al.[25]	2008	Canada	5279	68.8	10.3	472
Chaudhary et al.[26]	2008	Canada	100	69.7	1.9	2
Sando et al.[27]	2015	Canada	414	68.9	12.3	11
Rhee et al.[28]	2018	Canada	17,243	67.1	1	241
Demcoe et al.[29]	2019	Canada	2815	64.7	3.2	63
Khoshbin et al.[30]	2019	Canada	450	67.8	7.4	14
Teeter et al.[31]	2019	Canada	50	69.2	2	4
Garceau et al.[32]	2020	Canada	390	64.6	4	43
Shen et al.[33]	2009	China	68	61	5.9	1
Luo et al.[34]	2022	China	200	68.8	5	3
Wang et al.[35]	2022	China	120	65.9	8.6	1
Gaillard et al.[36]	2016	France	4189	70.4	3.3	87
Putman et al.[37]	2018	France	263	61	9.3	18
Erivan et al.[38]	2019	France	202	70.8	15	14
Schnurr et al.[39]	2012	Germany	1121	68	2.9	32
Hotfiel et al.[40]	2017	Germany	72	62	10.3	3
Fuchs et al.[41]	2018	Germany	79	59	5.8	9
Iosifidis et al.[42]	2014	Greece	100	70	9.5	3
Bouras et al.[43]	2017	Greece	206	67.8	13.2	17
Innocenti et al.[44]	2014	Italy	87	58.8	11.3	2
Rossi et al.[45]	2020	Italy	72	66	10	3
Giustra et al.[46]	2023	Italy	128	71.5	12.5	4
Nakamura et al.[47]	2014	Japan	505	68.8	12.4	20
Ueyama et al.[48]	2020	Japan	257	76,2	10.1	10
Schepers et al.[49]	2012	South Africa	154	67.5	5	3
Bae et al.[50]	2012	South Korea	3014	63.4	10.2	156
Lee et al.[51]	2013	South Korea	106	67	5.1	4
Kim et al.[52]	2014	South Korea	888	66.5	12.2	11
Kim et al.[53]	2016	South Korea	1190	53.3	12.6	42
Yang et al.[54]	2017	South Korea	113	66.9	10.1	5
Kim et al.[55]	2017	South Korea	364	65,6	11	5
Park et al.[56]	2018	South Korea	334	70.3	10	10
Oh et al.[57]	2018	South Korea	496	66.4	9.3	10
Kim et al.[58]	2021	South Korea	190	65	20.3	5
Kim et al.[59]	2021	South Korea	268	69.1	11.1	14
Baek et al.[60]	2021	South Korea	585	67.5	11.2	16
Baek et al.[61]	2022	South Korea	171	60.3	12.5	12
Lee et al.[62]	2023	South Korea	855	71.5	11.9	30
Munzinger et al.[63]	2010	Switzerland	411	67	5.1	12
Nouta et al.[64]	2012	The Netherlands	104	67	11	3
Kievit et al.[65]	2013	The Netherlands	807	67	3.6	27
Willems et al.[66]	2020	The Netherlands	408	68.4	5	18
van Ooij et al.[67]	2022	The Netherlands	1271	65.5	20.5	128
Clayton et al.[68]	2006	UK	212	67	5.1	6
Keenan et al.[69]	2019	UK	249	66.7	10	14
Clark et al.[70]	2021	UK	127	65.2	5	3
Brown et al.[71]	2021	UK	2172	68	5	62
Farhan-Alanie et al.[72]	2023	UK	199	65.1	10	11
Boettner et al.[73]	2016	USA	181	67	3.3	5
Chan et al.[74]	2017	USA CA	30	59.1	5.1	3
Varshneya et al.[75]	2022	USA CA	333,054	62.7	5	4108
Odland et al.[76]	2011	USA IA	67	48.5	12.4	11
Meding et al.[77]	2010	USA Indiana	619	70.4	11.9	8
Faris et al.[78]	2015	USA Indiana	10,843	69.2	8.1	44
Martin et al.[79]	2016	USA Minnesota	28,667	68	10.1	1570
Houdek et al.[80]	2017	USA Minnesota	11,653	71	8	724
Kremers et al.[81]	2014	USA MN	16,584	68.8	9.4	1180
Meftah et al.[82]	2012	USA NY	138	69.2	10	3
Deshmukh et al.[83]	2016	USA NY	486	64.9	3.5	10
Moussa et al.[84]	2017	USA NY	439	71.3	2	11
Dayan et al.[85]	2020	USA NY	404	65	6.2	21
Singh et al.[86]	2023	USA NY	133	61.5	2.4	6
Siljander et al.[87]	2023	USA NY	282	65.7	5	4
Yacovelli et al.[88]	2021	USA PA	5970	66.4	3.6	26
Bertin[89]	2005	USA UT	251	69.2	5.9	3
Peters et al.[90]	2014	USA UT	468	62	3.5	28
Pelt et al.[91]	2019	USA UT	141	64	3	19

### Clinical studies on revision rates of unicompartmental knee arthroplasty

We included twenty-six studies with 28,679 unicompartmental knee arthroplasties from 2009 to 2023 in our review, revealing 140,064.6 CY (Table 2).

**Table 2:** Data of clinical studies portraying unicompartmental knee arthroplasty

Authors	Year of publication	Country	Total UKA	Mean age (y)	Mean follow-up (y)	Revisions UKA (n)
Clark et al.[92]	2010	Australia	398	63.5	3.6	15
de Grave et al.[93]	2018	Belgium	460	66	5.5	11
Burnett et al.[94]	2014	Canada	467	69.3	6.1	38
Xu et al.[95]	2017	China	64	59	7.2	6
Knifsund et al.[96]	2017	Finland	294	67	8.7	53
Lustig et al.[97]	2009	France	172	72.2	5.2	11
Chatellard et al.[98]	2013	France	559	69.5	5.2	14
Sébilo et al.[99]	2013	France	944	70	5.2	17
Batailler et al.[100]	2019	France	160	68.5	1.8	11
Mergenthaler et al.[101]	2021	France	391	66.9	26.3	29
Heyse et al.[102]	2011	Germany	163	67.5	4.6	15
Heyse et al.[103]	2012	Germany	223	53.7	10.8	15
Maritan et al.[104]	2023	Italy	95	61.2	7.8	5
Woo et al.[105]	2022	Singapore	242	61	10	15
Song et al.[106]	2016	South Korea	68	64	9	3
Song et al.[107]	2019	South Korea	50	60.8	12	11
Lee et al.[108]	2022	South Korea	21,194	60.41	4	1390
Sever et al.[109]	2019	Turkey	133	65.5	10.5	21
Forster-Horváth et al.[110]	2016	UK	236	68.2	7.3	20
Chowdhry et al.[111]	2017	UK	265	51.7	7.7	6
Kennedy et al.[112]	2018	UK	1,000	66.6	10	52
Saenz et al.[113]	2010	USA	144	72	3	16
Edmiston et al.[114]	2018	USA IL	65	61.3	6.9	4
Kazarian et al.[115]	2020	USA Missouri	253	62.9	3.7	36
Berend et al.[116]	2012	USA OH	132	68	3.3	1
Hamilton et al.[117]	2010	USA Virginia	507	66	3.3	26

### Registers

National arthroplasty registers were accessed through the international registry network NORE (Network of Orthopedic Registries of Europe), which is a standing committee of EFORT (European Federation of National Associations of Orthopedics and Traumatology) [118]. Every register was included in their most recent presented edition. Registers had to provide data on total arthroplasty numbers (UKA and TKA), total revision numbers, sex, mean age, fixation, observed time period and covered country. Only registers who documented at least 90% of executed arthroplasties. Reports covering regional arthroplasty numbers or with insufficient data were excluded. Of 31 screened registers, a total of seven were included (Fig. 1). National arthroplasty registers from New Zealand, Sweden, Slovakia, Portugal, and Switzerland, covering different timeframes within 2003 to 2022, met the inclusion criteria and are represented in Table 3.

**Table 3:** Data of national registers regarding total knee arthroplasty and unicompartmental knee arthroplasty

Registry	Country	Total UKA	Revisions UKA	Mean Age UKA (y)	Total TKA	Revisions TKA	Mean age TKA (y)
Portuguese Arthroplasty Register May 2010[119]	Portugal	67			4,018	291	68.4
Swiss National Hip & Knee Joint Registry Report 2022[120]	Switzerland	15,364		64.5	91,129	12,309	69.5
New Zealand Orthopaedic Association Registry [121]	New Zealand	16,891	1,474	66.1	143,501	5,224	68.3
THE SWEDISH KNEE ARTHROPLASTY REGISTER – ANNUAL REPORT 2020 – PART I [122]	Sweden	7,690	1,562		127,060	4,691	
Australian Orthopaedic Association National Joint Replacement Registry[123]	Australia	70,925	4,813	65.4	886,536	26,004	68.4
THE SWEDISH KNEE ARTHROPLASTY REGISTER – ANNUAL REPORT 2020 – PART II[122]	Sweden	1,820	152		14,967	687	
Slovak Arthroplasty Register[124]	Slovakia				10,772	411	

### Outcome measures

The main aim of this review was to compare TKA and UKA revision rates amongst national registers and studies. To counterbalance different follow-up times and to make studies and registers more comparable, we calculated the revision rate and “revisions per 100 observed component years (CY)” [14, 15, 125, 126] when possible. This enables comparison of study data without the influence of follow-up times and cohort size.

Component years are calculated by mean follow-up time (in years) multiplied by the number of primary arthroplasties at the mean follow-up time. Therefore, longer mean follow-up times and larger numbers of primary arthroplasties have higher statistical power than studies with smaller groups and shorter follow-up.

In order to determine the revisions per 100 observed component years, the total number of revisions is firstly divided by the CY. Secondly, this quotient is multiplied by 100. Using this method, revision rates are made more quantifiable amongst studies and registers [14, 15, 125, 126].

Additionally, we compared revision rates in registers and mean revisions per 100 observed CY in TKA and UKA in studies, calculating the relative difference between revision rates of TKA and UKA. Correlation of revision rates and mean revisions per 100 observed CY in TKA and UKA were explored through linear regression computation.

Statistical analysis was executed using Microsoft Excel and IBM SPSS Statistics 21 (SPSS Inc., Chicago, IL).

## Results

### Clinical studies on survival rate of TKA

Seventy-one clinical studies published from July 2005 to September 2023 have met our inclusion criteria. In total 461,387 total knee arthroplasties and 9499 revisions were registered. Patient age was 64.2 on average. The mean follow-up time was 5.7 years revealing in 2,695,243.4 component years recorded. This exposes an average of 0.71 revisions per 100 component years and a mean revision rate of 3.35% amongst studies with a mean follow up of 5.7 years. Typical reasons for revisions were infection, stiffness, instability, trauma and septic or aseptic loosening. Full results are shown in Table 4.

**Table 4:** Results of calculations regarding total knee arthroplasty in clinical studies

Country	Total TKA	Revisions TKA (n)	Revision rate	Mean follow-up	Observed component years TKA (CY) (n)	Rev/CY x 100 (TKA)	Mean age (y)
China	388	5	1.28865979	6.2556701	2427.2	0.21557271	66.5360825
Italy	287	9	3.1358885	11.5090592	3303.1	0.29003492	66.2703833
South Korea	8574	320	3.73221367	11.1874831	95921.48	0.33905449	64.1784784
Japan	762	30	3.93700787	11.6209974	8855.2	0.35238344	71.285958
South Africa	154	3	1.94805195	5	770	0.38961039	67.461039
Belgium	245	11	4.48979592	11	2695	0.40816327	68.1012245
UK	2959	96	3.2443393	5.76716233	17065.0333	0.45146634	67.5048665
Greece	306	20	6.53594771	11.9908497	3669.2	0.47048668	68.5189542
Australia	1553	18	1.15904701	7.04007298	10933.2333	0.47183476	74.821217
Switzerland	411	12	2.91970803	5.09338462	2093.38108	0.57323533	67
France	4654	119	2.55694027	4.14518908	19291.71	0.6099024	69.8861624
The Netherlands	2590	176	6.7953668	12.4312355	32196.9	0.51284711	66.4790734
Total	461387	9499	3.35178146	5.6914844	2695243.37	0.712708	64.1634087
USA	410410	7784	1.89663995	5.68569207	2333464.88	0.91112994	63.7858367
Germany	1272	44	3.4591195	3.46041667	4401.65	1.12781742	67.1014151
Canada	26822	852	3.17799568	3.41080612	91484.6417	1.32466046	67.1870032

### Register data on TKA

The assessment of international register datasets resulted in seven registers originating from six countries, as shown in Table 5. Throughout 1,277,983 primary TKA and 49,617 revisions were listed. This concluded a pooled revision rate of 5.63% amongst register covering timeframes within 2003 to 2022. Sweden showcases itself with two datasets. The first one is covering the years 2009–2018 and the second one the year 2019.

**Table 5:** Results of calculations featuring total knee arthroplasty in national registers

Registry	Country	Total TKA	Revisions TKA	Proportion (Revisions TKA/total TKA) %
Australian Orthopaedic Association National Joint Replacement Registry	Australia	886536	26004	2.93321422
New Zealand Orthopaedic Association Registry	New Zealand	143501	5224	3.64039275
THE SWEDISH KNEE ARTHROPLASTY REGISTER–ANNUAL REPORT 2020–PART I	Sweden	127060	4691	3.69195656
Slovakisches Arthroplasty Register	Slovakei	10772	411	3.81544746
THE SWEDISH KNEE ARTHROPLASTY REGISTER – ANNUAL REPORT 2020–PART II	Sweden	14967	687	4.59009822
Total		1277983	49617	5.63153491
Portuguese Arthroplasty Register May 2010	Portugal	4018	291	7.24240916
Swiss National Hip & Knee Joint Registry Report 2022	Switzerland	91129	12309	13.507226

### Clinical studies on survival rate *of UKA*

There were twenty-six studies covering unicompartmental knee arthroplasty from February 2009 to November 2022 that matched our inclusion criteria. Overall, 28,679 unicompartmental knee arthroplasties and 1841 revisions in patients with a mean age of 61.89 years were recorded and results were portrayed in Table 6. With a mean follow-up of 4.9 years, 140,064.6 component years were traced. Out of 100 observed component years, 1.3 revisions were registered, along with a mean revision rate of 7.67% and a mean follow up of 4.9 years. Infection, instability, bearing dislocation, malpositioning, loosening, osteoarthritis, wear pain and trauma were characteristic reasons for revision.

**Table 6:** Results of calculations illustrating unicompartmental knee arthroplasty in clinical studies

Country	Total UKA	Revisions UKA (n)	Revision rate	Mean follow-up	Observed component years UKA (CY) (n)	Rev/CY x 100 (UKA)	Mean age (y)
Belgium	460	11	2.39130435	5.5	2530	0.43478261	66
Singspore	242	15	6.19834711	10	2420	0.61983471	61.0198473
UK	1501	78	5.19653564	9.17707972	13774.7967	0.65652002	64.220986
Italy	95	5	5.26315789	7.75763158	736.975	0.67844907	61.1715789
Australia	398	15	3.76884422	3.58333333	1426.16667	1.05177048	63.5
France	2226	82	3.68373765	8.63485924	19221.1967	1.22115831	69.3912848
China	64	6	9.375	7.2	460.8	1.30208333	59
Total	28679	1841	7.67147048	4.88387431	140064.631	1.30486561	61.8928046
Germany	386	30	7.77202073	8.18186528	3158.2	1.3116768	59.5274611
South Korea	21312	1404	6.58783784	4.01889255	85650.638	1.32323175	60.4223696
Canada	467	38	8.13704497	6.08333333	2840.91667	1.33759643	69.3
Turkey	133	21	15.7894737	10.5	1396.5	1.5037594	65.5
USA	1101	83	7.53860127	3.5	3890.64167	2.05154726	66.0346957
Finland	294	53	18.0272109	8.7	2557.8	2.07209321	67

### Register data *on UKA*

Data concerning UKA was illustrated in four registers, two originating from Sweden (2009–2018 and 2019). Throughout 97,326 unicompartmental knee arthroplasties and 8001 revision surgeries were recorded. This is resulting in a pooled revision rate of 11.04% amongst registers covering periods within 2003–2022 (Table 7).

**Table 7:** Results of calculations depicting unicompartmental knee arthroplasty in national registers

Registry	Country	Total UKA	Revisions UKA	Proportion (Revisions UKA/total UKA) %
Australian Orthopaedic Association National Joint Replacement Registry	Australia	70925	4813	6.78604159
THE SWEDISH KNEE ARTHROPLASTY REGISTER–ANNUAL REPORT 2020–PART II	Sweden	1820	152	8.35164835
New Zealand Orthopaedic Association Registry	New Zealand	16891	1474	8.72654076
Total		97326	8001	11.0440811
THE SWEDISH KNEE ARTHROPLASTY REGISTER–ANNUAL REPORT 2020–PART I	Sweden	7690	1562	20.3120936

### TKA and UKA revision rates in studies

Sufficient study data on revision rates for TKA and UKA were documented for ten nations. When looking at TKA, we recorded 2,580,987.9 observed component years, with a mean revision rate of 6.15 revisions per 100 CY at a mean age of 67.5 years. With 133,690 observed component years UKA showed a mean revision rate of 1.14 revisions per 100 CY at a mean patient age of 63.9. As a result, there were on average 2.29 times higher revision rates and 1.85 times more revisions per 100 CY in the UKA cohorts than in the TKA cohorts, with similar divergence between countries.

**Fig. 2:**
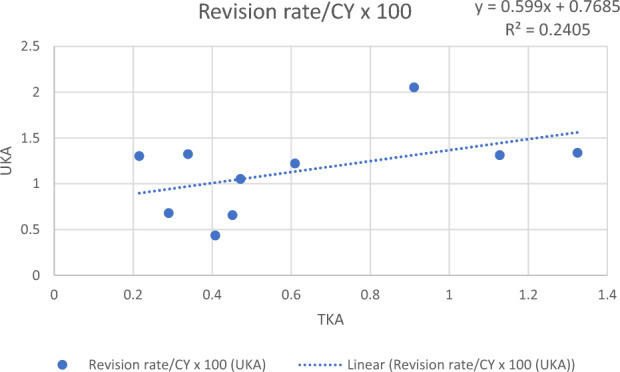
Chart of linear regression of revision rate/component years × 100 of total knee arthroplasty and unicompartmental knee arthroplasty in clinical studies by countries

A positive correlation of pooled revision rates of TKA with UKA in studies was found. Considering a broad array of data was used, with varying numbers of studies depicting region specific revision rates, a coefficient of determination of R^2^ = 0.2405 is indicating linear correlation (Fig. 2).

### TKA and UKA revision rates in registers

There were solely four registers reporting on TKA and UKA at the same time, with Sweden being represented twice (2009–2018 and 2019). While the mean revision rate for TKA was at 3.71%, the mean revision rate for UKA was at 8.73%, being 2.38 times as high and showing similar variation amongst countries.

**Fig. 3:**
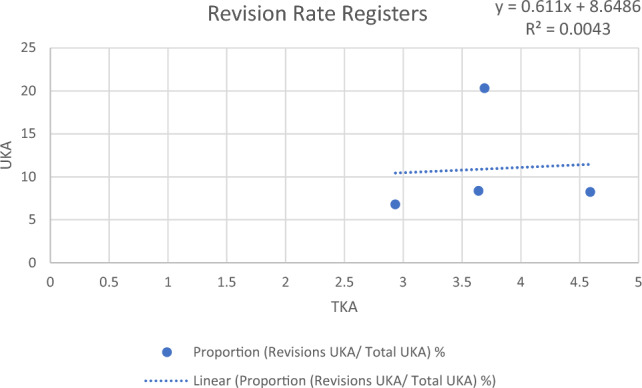
Chart of linear regression of revision rates of total knee arthroplasty and unicompartmental knee arthroplasty in national arthroplasty registers

Only four registers reporting on three countries provided data for both, TKA and UKA, resulting in a low coefficient of determination of R^2^ = 0.0043. Nevertheless, considering the large number of cases featured in arthroplasty registers, a positive correlation of TKA and UKA revision rates could be found (Fig. 3).

## Discussion

The most important finding of this study was a positive correlation of revision rates of TKA and UKA in different regions in registers and studies, affirming our hypothesis. Studies dealing with UKA revealed 2.29 times higher revision rates in comparison to TKA. Register data continue this trend by presenting 1.96 times higher revision rates for UKA than for TKA.

Mean revision rates in registers (5.63% TKA, 11.04% UKA) were about 1.56 times as high as pooled revision rates described in studies (TKA: 3.35% and 0.71/100CY; UKA: 7.67% and 1.3/100CY UKA). On that note a certain degree of comparability is given. This correlation could be registered in a few countries represented in studies and registers such as TKA in Switzerland, where revision rate in registers was 5.63% and studies showed 2.92% (0.57 rev/100CY). Same relation was observed in revision numbers of UKA in Australia (registers 6.79% vs. studies 3.77% & 1.05 rev/100CY). However, revision rates of TKA in Australia showed divergent behaviour (registers 13.51% vs. studies 1.16% & 0.57 rev/100CY).

Registers represent the national average values, while studies record smaller cohorts limited to one or more defined centers. High volume centers and surgeons, who specialise in these procedures show significantly lower revision rates than low volume surgeons and centers [127]. With higher volume surgeons and centers publishing more papers, lower revision rates as described in studies appear comprehensible.

Apart from center and surgeon volume, surgeon experience and more recent investigations also had great influence on the reported revision rate. The Swedish Arthroplasty Register Part II covering solely the year 2019 is showing values near the pooled revision rate for TKA and UKA, while Part I of the Swedish Arthroplasty Register dealing with the years 2009–2018 is reporting much higher revision rates for UKA.

Higher revision rates for UKA don’t necessarily depict bad function or worse outcomes than TKA. Orthopaedic surgeons experiencing some sort of complications after UKA implantation tend to show lower threshold for revision of aseptic UKA than TKA. The reason for this is suspected in TKA being the revision for UKA and surgeons having more experience in the implantation of TKA in respect to UKA [128–130]. Revision of UKA through TKA show satisfying results, although is accompanied by greater bone loss, more augments and thicker polyethylene components, while providing similar revision rates, complications and hospital stay than primary TKA [131]. UKA being revised using another UKA is rarely performed. Finally, revision threshold for UKA is significantly lower than for TKA [129].

Study patients receiving TKA show a mean age of 64.2 years and an average of 5.7 years of follow up. Participants in papers representing UKA display lower age (61.9 years) with shorter follow-up time (4.9 years). Registers show a similar trend for mean patient age, with UKA patients being younger.

Some limitations need to be noted. This analysis includes a great amount of study and register data, whereby a vast array of cases is covered. Unfortunately, many data sets are incomplete, preventing inclusion in studies of any kind to some extent. This is resulting in studies and national register that cannot be included in studies because of a lack of information, showing that quality of meta-analysis is dependent on the quality of data presented. Additional limitation was poor accessibility, indistinct display of information and foreign language of registers, lowering reproducibility and impeding analysis. Furthermore, since no patient-reported outcome measures were listed, no quality of clinical outcome of UKA or TKA can be demonstrated.

In order to provide comparability and reproducibility, more nations should conduct accessible arthroplasty registers supplying sufficient data in English language. In succession, future studies on revision rates could be compared to international registry data, making it easier to elaborate differences and promoting research on knee arthroplasty.

## Conclusion

A positive correlation of revision rates of TKA and UKA in studies and registers was found. Revision rates of UKA comparted to TKA were 2.29 times higher in clinical studies and 1.96 times higher in registers. Revision rates in registers were about 1.56 times higher than presented in clinical studies.

## Data Availability

Additional data is available on request.
